# Safety and efficacy of hairy scalp donors in thick split-thickness skin grafting: Healing and complications at donor sites

**DOI:** 10.1016/j.jpra.2024.12.007

**Published:** 2024-12-19

**Authors:** Seung Ho Lee, Suk Joon Oh, Chanho Jeong, Kunyong Sung, Jong Dae Kim, Jeong Tae Kim

**Affiliations:** 1 Department of Plastic and Reconstructive Surgery, Kangwon National University Hospital, 156 Baekryeong-ro Chuncheon Kangwon-do, 24289, Republic of Korea; 2 Department of Burn Reconstructive Surgery, Daejeon Hwa Hospital, 39, Dongdaejeon-ro, Dong-gu, Daejeon, Republic of Korea; 3 Department of Plastic & Reconstructive Surgery, Bestian Seoul Hospital, 382, Wangsimni-ro, Seongdong-gu, Seoul, Republic of Korea

**Keywords:** Scalp skin grafting, Wound healing, Ultrasonography, Histometry

## Abstract

**Background:**

We aimed to investigate how the thickness of removed skin grafts affects the healing time at the donor site, with a focus on the depth of the donor wound and the normal thickness of the scalp.

**Methods:**

We examined the outcomes of the donor sites of thick split-thickness skin grafts using hairy scalp skin in 102 Korean patients. We measured the thickness of the scalp donor skin using preoperative ultrasonography, histometric thickness of normal scalp skin in 61 patients, and histometric thickness of the thickest part of the grafted skin after surgery.

**Results:**

The mean normal ultrasound thickness of the scalp donors was 1.711 mm, with a mean histometric normal scalp thickness of 1.926 mm (61 cases), mean dermatome depth set of 0.569 mm (22/1000 inches, 22 mils), and mean histometric harvested skin thickness of 0.677 mm. The relationship between healing time and percentage of histometric graft thickness per ultrasonographic normal scalp skin thickness was statistically significant and correlated positively. Healing of the partial portion of the scalp was delayed in 9 donors due to infection and folliculitis. Scalp donor wounds healed during postoperative days 6 and 15, except for the ones with infected portions.

**Conclusion:**

Patients in this study successfully achieved scarless regenerative healing of their scalp donor wounds. Furthermore, adequate wound dressing can prevent alopecia and scarring of infected donor wounds. The results of this study offer valuable insights into the advantages of using scalp skin in grafts, underscoring its potential as a preferred option for achieving optimal regenerative healing.

## Introduction

Crawford^1^ first introduced the use of scalp skin as an unusual donor site for split-thickness skin grafts (SSGs) to treat extensive deep burns in children.

The harvested thick scalp SSG was 69% as thick as the normal scalp skin (0.035/0.051 inches), and interfollicular epithelization of the 0.035-inch-thick harvest was accomplished 11 days after the surgery at the scalp donor sites.^2^ Furthermore, scalp donor wounds with a mean of 0.018 inches (range, 0.016–0.022 inches) 2-layer dermatome depth sets were epithelized at a mean of 9.9 days after surgery (range, 7–14 days).^3^

We aimed to investigate how the thickness of the removed grafted skin, as a percentage of normal scalp thickness, affects the healing time at the donor site. We also examined the histological findings of the healing donor site and its associated complications.

## Methods

This retrospective study included 102 Korean patients who underwent hairy scalp SSG of ≥0.406 mm (16 mils) of a dermatome depth set and were hospitalized in our department.

Data on sex, age, ultrasound, histometric thicknesses of the normal scalp skin, and histometric graft thickness at the time of surgery were collected from the patient's medical records.

The postoperative variables included the donor wound healing time, its statistical relationship with healing time, the percentage of variable thickness related to the donor wound, and the incidence of complications.

The incidences of late and early complications were reviewed after 1 year of follow-up.

### Surgical technique

The scalp was shaved and sterilized with a povidone solution. Subsequently, 1:300,000 adrenaline mixed with physiological saline (NaCl 0.9%) was injected into the subgaleal space to create a cushion, facilitating the harvest of a broad region of skin. Scalp SSGs were harvested using a Zimmer Air Dermatome (Zimmer Inc., Warsaw, IN, USA). Hemostasis was achieved using a temporary gauze dressing soaked in a solution containing 10 mg adrenaline in 1 L NaCl 0.9%. After hemostasis, a Mepitel sheet (Mölnlycke Health Care, Gothenburg, Sweden) was tightly fixed to the donor wound with staples, covered with absorbent gauze, and secured using an elastic bandage and stocking.

### Assessment

Preoperative ultrasonography (Esaote MyLab One, Genoa, Italy) was used to measure skin thickness at the donor site. We confirmed the histometric thickness of the thickest portion of the scalp graft skin and that of the normal scalp skin near the donor site. The scalp donor healing process was confirmed by the histological examination of a punch biopsy of the donor wounds. Donor site healing was defined as nearly ≥95% epithelialization at the donor site.

The postoperative variables included the healing time of the donor wound, the statistical relationship between the healing time and percentage of variable thickness related to the donor wound, and the incidence of early and late complications.

We treated 102 patients with thick scalp SSGs, setting the dermatome depth to ≥0.406 mm to reduce the contracture of the recipient graft and morbidity of the donor site. Our study data included preoperative ultrasound scalp skin thickness, postoperative histometric graft thickness, and dermatome depth set (102 patients), including histometric hairy normal scalp skin thickness with a 3-mm punch biopsy (61 patients) during surgery.

### Statistical analysis

For proper statistical analysis, the parameters of the thickness of the dermatome depth set and graft tissue were changed in inches and mm to mils. A paired t-test was performed to investigate the differences between normal scalp skin histology and sonographic thickness. One-way analysis of variance (ANOVA) was conducted to examine the relationship between healing time and the dermatome depth set. Simple linear regression analysis was performed to investigate the relationship between healing time and percentage of dermatome depth set in normal scalp skin thickness, healing time and percentage of histometric graft thickness in histometric normal scalp skin thickness, and healing time and percentage of histometric graft thickness in ultrasound normal scalp skin thickness. All statistical analyses were performed using the R Statistical Software (version 4.0.3; R Foundation for Statistical Computing, Vienna, Austria).

## Results

102 patients, including 70 males and 32 females with a mean age of 33.3 ± 23.8 years (range: 0.8–78 years) underwent the surgery.

Grafted skin thickness was measured histometrically in 102 patients. The mean dermatome depth was 22 ± 4.478 mils (range, 16–32 mils), and the mean histometric graft thickness was 27 ± 6.871 mils (range, 11–50 mils). The mean thickness difference of each case was 5.2 mils (95% CI, 3.9–6.4). In the paired t-test, the difference between the 2 types of thickness was statistically significant (P < 0.001). The Pearson's correlation coefficient was 0.481 (Table 1).

**Table 1:** Paired t-test between dermatome depth set and histometric thickness of graft (1/1000 inches, 1 mil).

	N	Mean	SD	Range	Means of the differences (95% CI)	P value (Paired t-test)	*r*
Dermatome depth set	102	22	4.5	16–32	5 (3.9–6.3)	<0.001	0.494
Graft thickness (histometric)	102	27	6.8	16–50			

N, number; SD, standard deviation; CI, confidence interval; *r*, Pearson correlation coefficient.

The dermatome depth set values were not statistically correlated with healing time in 1-way ANOVA (Table S1).

The mean percentage of dermatome depth set to ultrasound scalp skin thickness of the 102 patients was 33.18% ± 9.06% (range, 16.7%–61.5%). The relationship between the percentage of dermatome depth set thickness in normal ultrasound skin thickness and healing time was not statistically correlated with healing time in a simple linear regression analysis (r^2^ = 0.009, SE = 0.022, t-value = 0.975, P = 0.332) (Figure S1).

The mean percentage of histometric graft thickness to the histometric thickness of normal scalp skin in 61 patients was 37.82% ± 12.50% (range, 20%–80%). The relationship between healing time and percentage of histometric graft thickness per histometric normal scalp skin thickness is statistically significant; however, the degree of correlation is low. The simple regression equation was healing time (days) = 7.639+0.050 × percentage thickness (%) (r^2^ = 0.094, SE = 0.020, t-value = 2.469, P = 0.017) (Figure S2).

**Figure 1:**
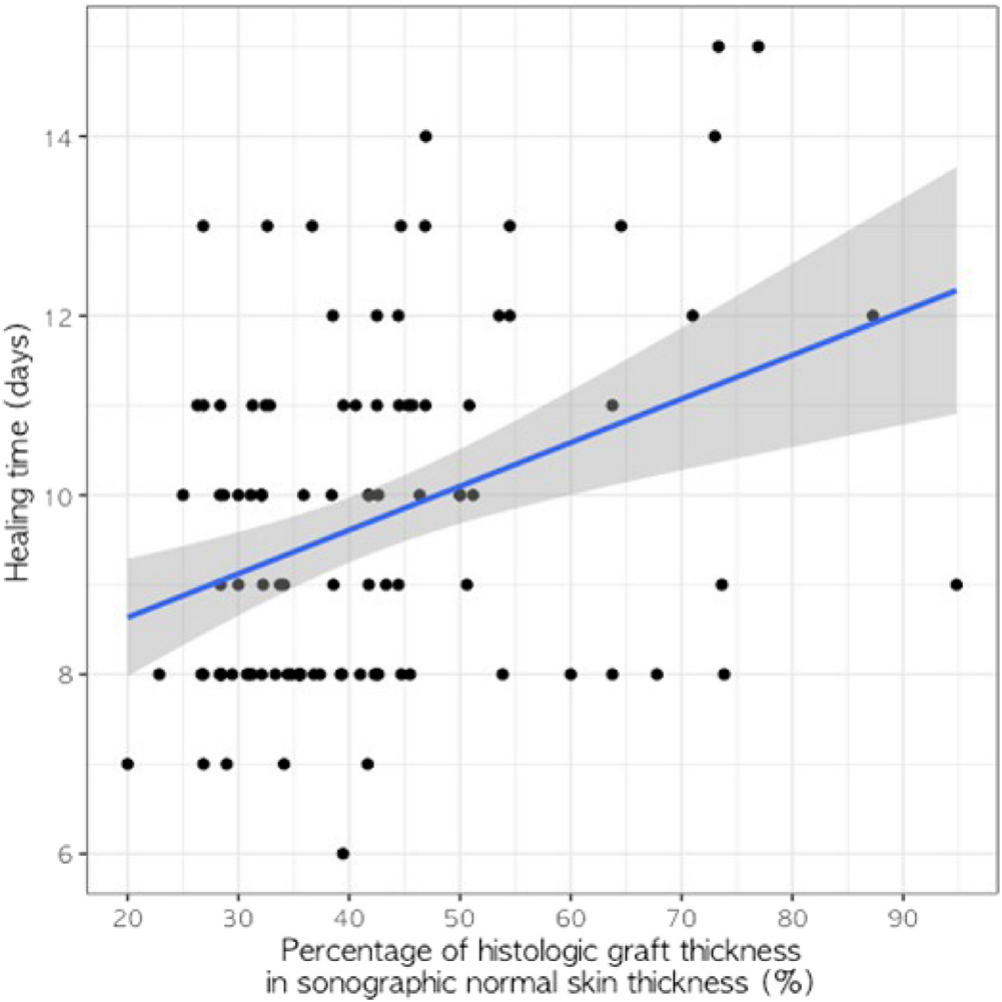
Relationship between percentage of histologic graft thickness in sonographic normal skin thickness and healing time.

The mean percentage of the histometric thickness of scalp SSGs to ultrasound scalp skin thickness in 102 patients was 41.38% ± 14.04% (range, 20%–94.8%). The relationship between healing time and percentage of histometric graft thickness per ultrasound normal scalp skin thickness was statistically significant and positively correlated. The simple regression equation was healing time (days) = 7.400+0.055 × percentage thickness (%) (r^2^ = 0.158, SE = 0.013, t-value = 4.332, P < 0.001) (Figure 1).

The scalp donor wounds healed during postoperative days 6 and 15, except for the infected portion.

**Figure 2:**
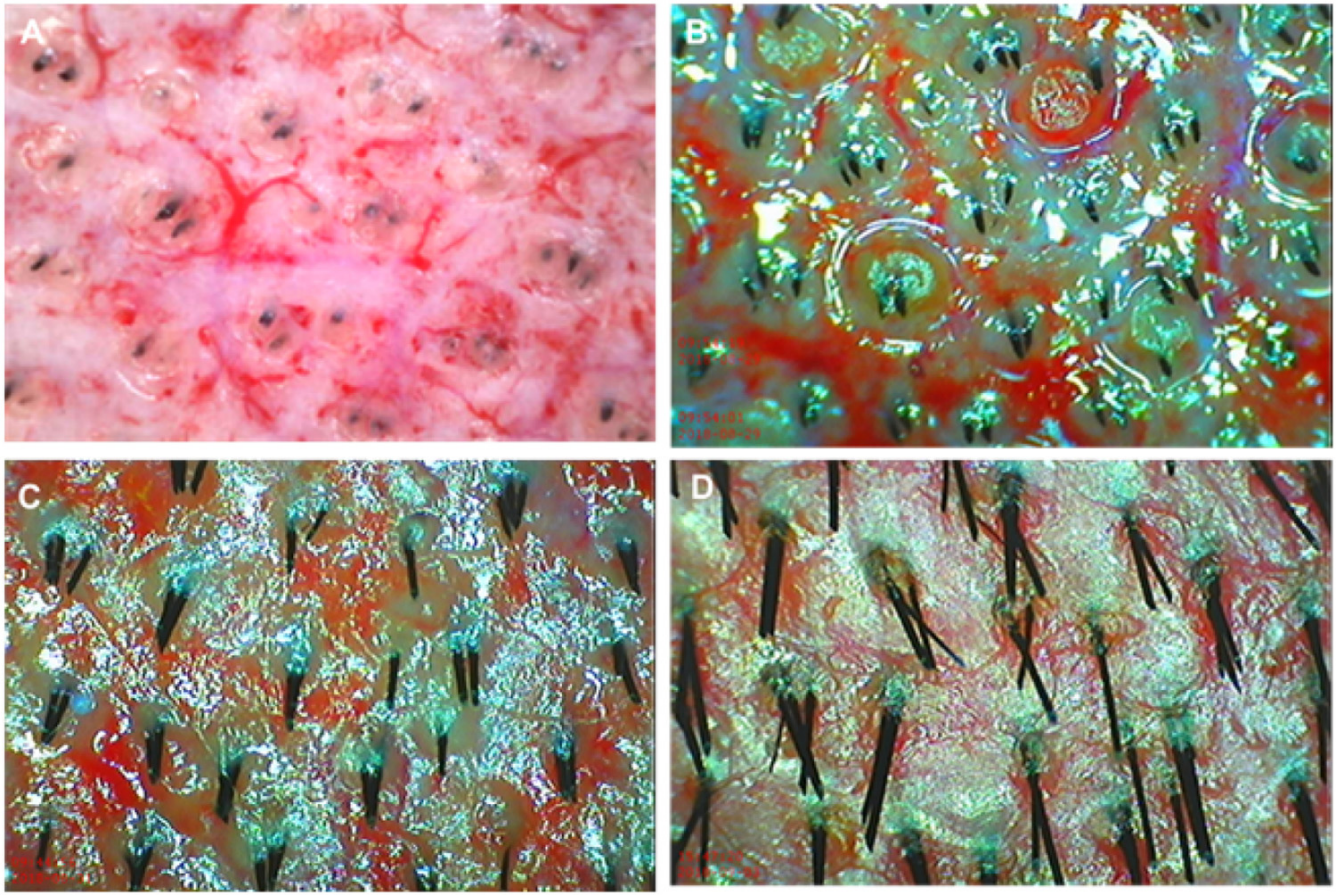
Dermoscopic findings ( × 15) of scalp donor healing. **(A)** Donor wounds just after harvesting 0.02-inch thickness graft. **(B)** Whitish epithelial regeneration from the cut hair unit at postoperative day 1. **(C)** Epithelial regeneration nearly covered the fibrin-coated wound at postoperative day 3. **(D)** Epithelial regeneration completely covered the wounds on postoperative day 6.

Dermoscopic findings of the healing process of scalp donor wounds showed that whitish epithelium from the surrounding cut-hair unit appeared and spread centrifugally during the early postoperative period. If the interfollicular space was filled with whitish epithelium, the donor wound closed completely within 15 days of surgery (Figure 2).

**Figure 3:**
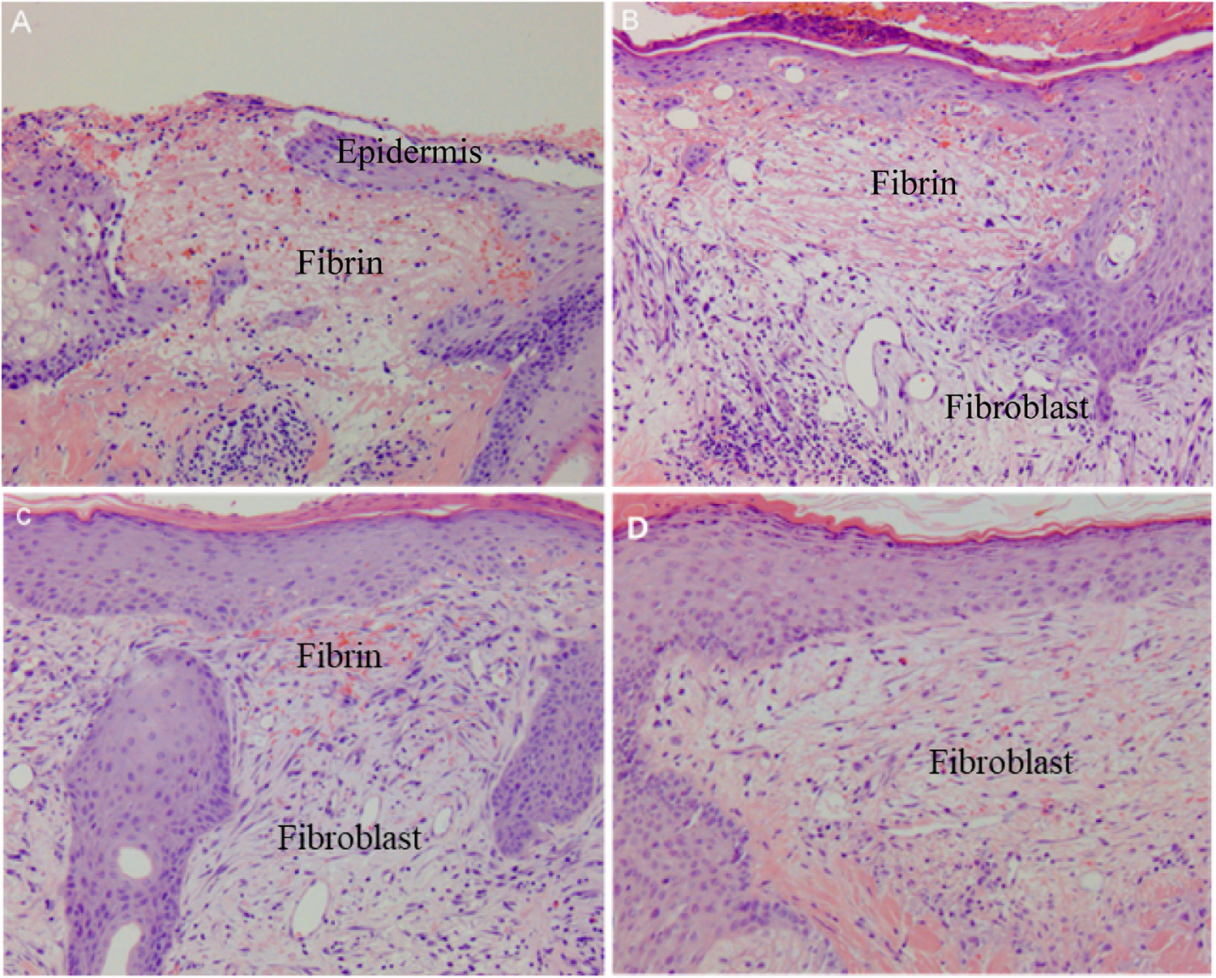
Histological findings of scalp donor wounds (HE stains, × 100). **(A)** Epithelial regeneration over the fibrin-coated wound at postoperative day 3. **(B)** Basket-weave-like fibroblast regeneration between wound bed and fibrin-coated layer at postoperative day 6. **(C)** Regenerative fibroblast with diminished fibrin tissue filled between wound bed and epidermal layer at postoperative day 8. **(D)** Regenerative fibroblast produced extracellular materials (collagen) at postoperative day 11.

Histological findings of the donor wound showed simultaneous basket-weave-like fibroblast cells under the regenerative epithelium, probably due to epithelial-mesenchymal interactions during the early period of wound healing. Postoperatively, basket-weave-like cells gradually produced collagen fibers from the border of the deep wound bed. Hairy scalp skin donors have suggested regenerative wound healing in restricted postnatal tissue. We examined the stem cell phenomenon of plasticity, focusing on recent observations of *in vivo* plasticity of dermal sheath cells (Figure 3).

Nine patients experienced delayed healing of the partial portion (mean, 38.1 days; range, 24–49 days) and of donor sites due to infection and folliculitis (mean, 25%; range, 5%–60%). Wound cultures reported multidrug-resistant *Acinetobacter baumannii* in 2 patients, methicillin-resistant *Staphylococcus aureus* (MRSA) in 2 patients, *Staphylococcus aureus* in 1 patient with concrete scalp deformity, and no growth in 4 patients. Donor wounds with delayed healing ultimately healed adequately with conservative dressing, except in 1 patient with alopecia.

**Figure 4:**
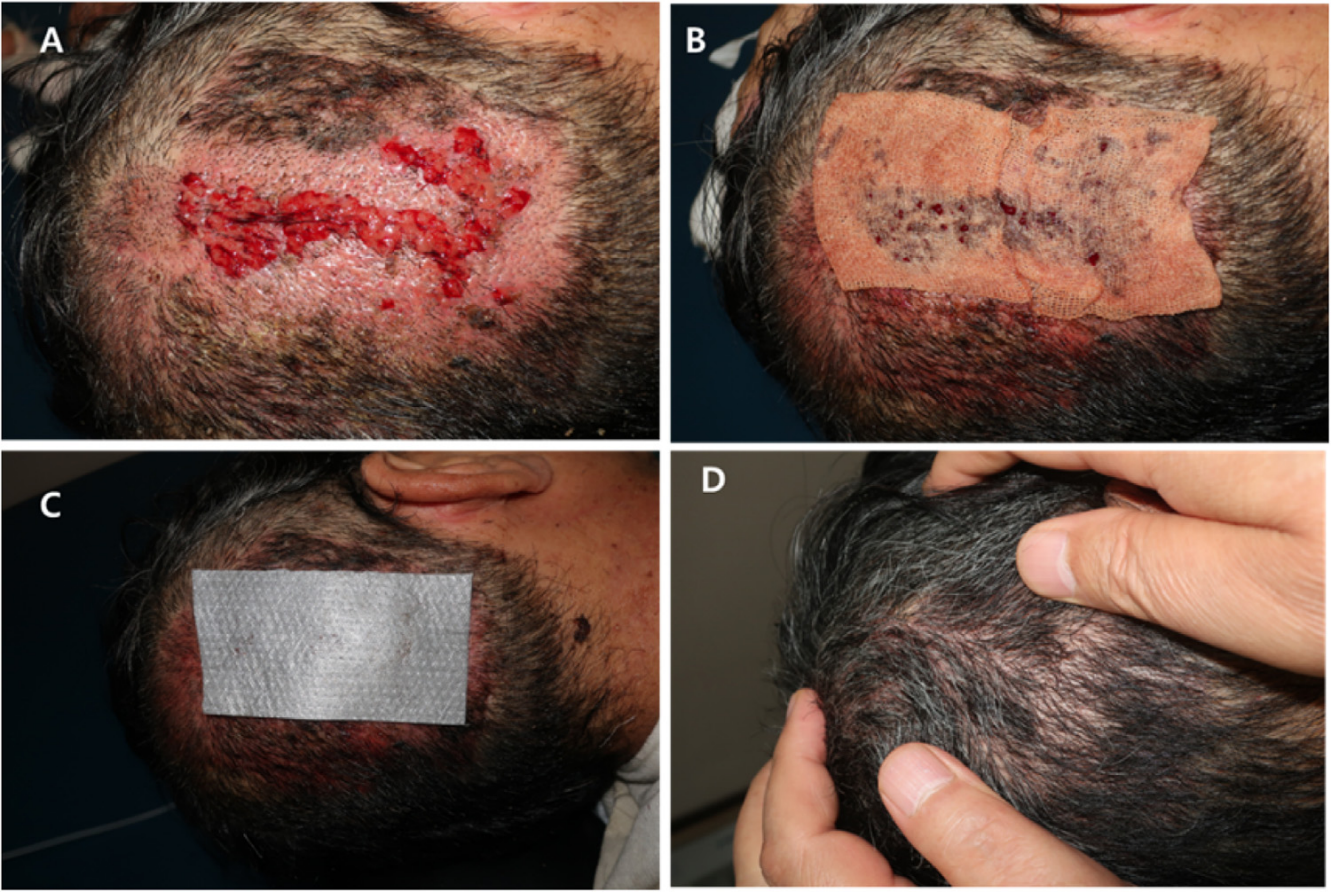
The early complication of scalp donors. **(A)** Concrete scalp deformity. **(B)** Cauterization with albothyl-soaked gauze. **(C)** Exsalt application. **(D)** Complete healing wound.

The primary treatment for folliculitis and scab formation was conservative, followed by rinsing the scalp with povidone solution and topical application of antiseptic ointment. This process was repeated daily until the folliculitis or scab formation was resolved. An albothyl solution (Celltrion Inc. Incheon, Republic of Korea) was used to clean the wound and for the cauterization of the granulation tissue, and Exsalt® dressing (Exciton Tech, Edmonton, AB, Canada) was used to treat the concrete scalp deformity due to infection (Figure 4).

**Figure 5:**
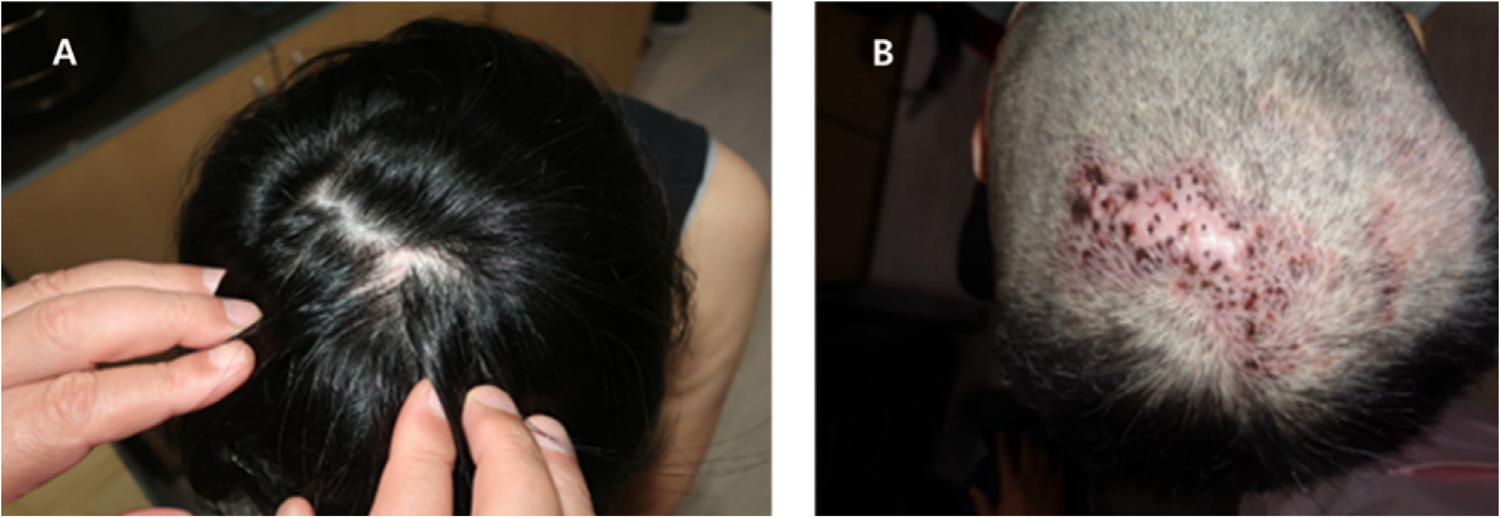
Late complications of scalp donor wounds. **(A)** Alopecia after the partially too thick harvested graft. **(B)** Tufted hair scalp after unrecognized folliculitis.

Late complications at the scalp donor site included alopecia in 2 patients and tufted scar deformity (2 × 5 cm in size) in 1 patient due to MRSA (Figure 5).

## Discussion

Among 27 articles of a systematic review of the scalp donor site for SSG, the depth set of the dermatome was < 0.016 inches (16 mils) in 17 articles, ≥ 0,016 inches in 7 articles, and no mention in 3 articles. In addition, the mean number of days of healing of the scalp donor site was < 10 days in 13 articles, between 10 and 14 days in 3 articles, >14 days in 2 articles, and no mention in 9 articles.^4^ Reportedly, the 0.035-inch-thick harvest scalp wound healed at 11 days postoperatively.^2^ The mean healing time of scalp donors was 9.9 days (range, 7–14 days), with a mean wound defect thickness of 0.018 inches (range, 0.016–0.022 inches).^3^

In the current study, we examined 102 patients with a thick SSG with ≥ 0.016 inches of dermatome depth set, owing to less contracture of the recipient graft and less morbidity of the donor site. Our study data included preoperative ultrasonographic scalp skin thickness, postoperative histometric graft thickness, and a dermatome depth set (102 patients), including histometric hairy scalp skin thickness with a 3-mm punch biopsy (61 patients) during surgery.

The wound depth of the scalp donor was predicted using the following 3 methods: percentage of dermatome depth set per ultrasonographic scalp skin thickness, percentage of histometric graft thickness per ultrasonographic scalp skin thickness, and percentage of histometric graft thickness per histometric scalp skin thickness. To predict the healing day of the scalp donor wound, a simple linear regression analysis was used to confirm the relationship between the healing time of the scalp donor wound and the above 3 methods. The relationship between healing time and wound depth of the scalp donor, which was related to the percentage of histometric graft thickness per ultrasonographic normal scalp skin thickness, was statistically significant and positively correlated. Nevertheless, all scalp donor wounds healed within 6–15 days postoperatively if wound infections did not occur.

Dermoscopic findings of the healing process of scalp donor wounds showed an appearance of whitish epithelium from the surrounding cut-hair unit on the first day after operation day and spread centrifugally during the early postoperative days. If the interfollicular space is filled with whitish epithelium, the donor wound closes completely within 6–15 days after surgery. These findings show early regenerative appearances of scalp donor wounds (Figure 2).

In wound beds after scalp SSG harvesting, regenerative healing can be induced without complications by maintaining the appropriate thickness of the reticular dermis without damaging the hair follicle. Although stem cells of the hair follicle bulge do not normally contribute to epidermal cells, if the epidermis is damaged, cells from the bulge and hair shaft are recruited to the epidermis and move linearly toward the center of the wound.^5^^,^^6^

Dermal fibroblasts undergo differentiation and lineage commitment to give rise to both the upper and lower dermal lineages. The dermal papilla, arrector pili muscle, dermal sheath, and papillary dermis of the scalp are derived from the upper dermal lineage.^7^ The dermal components of scalp hair follicles exhibit several stem cell properties, including regenerative potential, wound healing, and the ability to produce a functional dermis.^8^ Autologous transplantation of scalp punch grafts is a minimally invasive procedure that appears to be effective as a therapeutic tool for chronic venous leg ulcers and induces better healing than skin grafts harvested from non-hairy areas.^9^ There is great potential in exploring the role of hair follicles in wound healing and skin regeneration.^10^ Fibroblasts of the dermal papilla and dermal sheath of hair follicles are multipotent stem cells.^11^ Epidermal β-catenin activation stimulates the expansion of the upper dermal lineage in rendering wounds permissive to hair follicle formation of the scalp skin.^12^ Interactions between epithelial and mesenchymal cells influence hair follicle regeneration and wound healing.^13^

We examined the stem cell phenomenon of plasticity, focusing on recent observations of the *in vivo* plasticity of epidermal and dermal sheath cells.

In a systematic review, early complications of scalp donor sites included infections (7 patients), folliculitis (38 patients), concrete scalp deformity (18 patients), scab formation (38 patients), non-healing wounds (13 patients), and transient moth-eaten appearance (3 patients). The incidence of early complications at the scalp donor site was 3.82% (117 out of 3,062 patients). The incidence of late complications of alopecia occurred in 4.74% of patients after scalp skin harvest (145 out of 3,062).^4^

Concrete scalp deformities, scab formation, non-healing wounds, and transient moth-eaten appearance are associated with infection and folliculitis. In this study, the incidence of early complications of infection and folliculitis was 8.82% (9 out of 102 patients). Our high infection rates may be insightful evidence, as hospital-acquired infections are high and the scalp is densely populated with microbiologically diverse hair follicles, which may increase the risk of infection. The incidence of late complications of alopecia (2 patients) and tufted hair scalp (1 patient) was 2.94% (3 out of 102 patients).

In conclusion, thicker harvested grafts are estimated to have a longer healing time of the donor wound. Wound healing at the donor site begins around the epidermis and dermal sheath fibroblasts of the hair unit, probably due to epidermal-mesenchymal interaction. Hairy scalp skin donors have suggested regenerative wound healing in restricted postnatal tissue. Hairy scalp skin donors healed between 6 and 15 days postoperatively, except for infection or damaged hair units. Authors recommend hairy scalp skin as a safe donor site for thick skin grafting.

## Ethical approval

The study was approved by the Institutional Review Board of Bestian Seoul Hospital (IRB BMC No. 2024-05-008) and performed under the principles of the Declaration of Helsinki.

## Authors’ contributions

**S.J.O.** was responsible for conceptualization, data collection, and editing. **S.H.L.** was responsible for writing an original draft. **C.H.J.** and **K.Y.S.** were responsible for writing and reviewing. **J.D.K.** was responsible for statistical analysis. **J.T.K.** was responsible for resources.

## Patient consents

The patient provided written informed consent for the publication and use of his images.

## Conflict of interest

The authors declare that the article and its content were composed in the absence of any commercial or financial relationships that could be construed as a potential conflict of interest.

## Appendix Supplementary materials

Image, application 1

Image, application 2

Image, application 3
