# Supplementary material for: Safety and efficacy of hairy scalp donors in thick split-thickness skin grafting: Healing and complications at donor sites
Source: JPRAS Open. 2024 Dec 19;43:384–92. doi: 10.1016/j.jpra.2024.12.007 (PMC11782865; doi:10.1016/j.jpra.2024.12.007)
Supplement: Supplementary file 1 [file mmc1.docx]

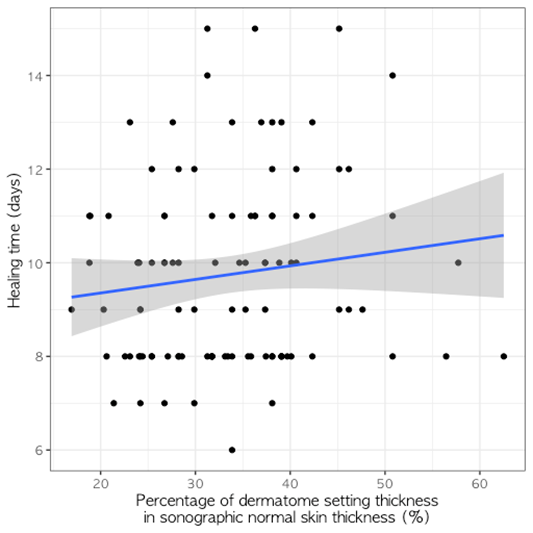


Figure S1. The relationship between the percentage of dermatome depth set thickness in sonographic normal skin thickness and healing time.
