# Supplementary material for: Safety and efficacy of hairy scalp donors in thick split-thickness skin grafting: Healing and complications at donor sites
Source: JPRAS Open. 2024 Dec 19;43:384–92. doi: 10.1016/j.jpra.2024.12.007 (PMC11782865; doi:10.1016/j.jpra.2024.12.007)
Supplement: Supplementary file 3 [file mmc3.docx]

| Table 2. Donor site healing time according to dermatome depth set | | | | |  |
| --- | --- | --- | --- | --- | --- |
| Dermatome depth set (inches) | N | Mean of healing time (days) | SD of healing time (days) | Range of healing time (days) | P value (One-way ANOVA) |
| Below 20/1000 | 26 | 10 | 2.38 | 7-15 | 0.741 |
| 20/1000 ~ 24/1000 | 46 | 9.65 | 1.89 | 6-14 |  |
| 25/1000 ~ 29/1000 | 18 | 9.33 | 1.61 | 8-13 |  |
| Above 30/1000 | 12 | 9.75 | 1.86 | 8-13 |  |
| N, number; SD, standard deviation | | | | | |
